# Clinical outcomes, quality of life, and costs evaluation of peritoneal dialysis management models in Shanghai Songjiang District: a multi-center and prospective cohort study

**DOI:** 10.1080/0886022X.2021.1918164

**Published:** 2021-04-29

**Authors:** Xiaoyan Ma, Min Tao, Yan Hu, Lunxian Tang, Jiasun Lu, Yingfeng Shi, Hui Chen, Si Chen, Yi Wang, Binbin Cui, Lin Du, Weiwei Liang, Guansen Huang, Xun Zhou, Andong Qiu, Shougang Zhuang, Xiujuan Zang, Na Liu

**Affiliations:** a Department of Nephrology, Shanghai East Hospital, Tongji University School of Medicine, Shanghai, China; b Emergency Department of Critical Care Medicine, Shanghai East Hospital, Tongji University School of Medicine, Shanghai, China; c Department of Urology, Shanghai East Hospital, Tongji University School of Medicine, Shanghai, China; d School of Life Science and Technology, Advanced Institute of Translational Medicine, Tongji University, Shanghai, China; e Department of Medicine, Rhode Island Hospital and Alpert Medical School, Brown University, Providence, RI, USA; f Department of Nephrology, Shanghai Songjiang District Central Hospital, Shanghai, China

**Keywords:** Peritoneal dialysis, management model, clinical outcomes, quality of life, costs evaluation

## Abstract

**Background:** The new Family-Community-Hospital (FCH) three-level comprehensive management aimed to improve the efficiency and scale of peritoneal dialysis (PD) to meet the increased population of end-stage renal disease (ESRD). Our study focused on the clinical outcomes, quality of life, and costs evaluation of this model in a multi-center and prospective cohort study.

**Methods:** A total of 190 ESRD patients who commenced PD at Shanghai Songjiang District were enrolled. According to different PD management models, patients were divided into the Family-Community-Hospital three-level management model (*n* = 90) and the conventional all-course central hospital management model (*n* = 100). The primary outcome was clinical outcomes of PD. The secondary outcomes were health-related quality of life (HRQOL) and medical costs evaluation.

**Results:** Compared to conventional management, community-based FCH management achieved a similar dialysis therapeutic effect, including dropout rate (*p* = 0.366), peritonitis rate (*p* = 0.965), patient survival (*p* = 0.441), and technique survival (*p* = 0.589). Follow-up data showed that similar levels of the renal and peritoneal functions, serum albumin, cholesterol and triglyceride, PTH, serum calcium, and phosphorus between the two groups (all *p* > 0.05). HRQOL survey showed that the FCH management model helped to improve the psychological status of PD patients, including social functioning (*p* = 0.006), role-emotional (*p* = 0.032), and mental health (*p* = 0.036). FCH management also reduced the hospitalization (*p* = 0.009) and outpatient visits (*p* = 0.001) and saved annual hospitalization costs (*p* = 0.005), outpatient costs (*p* = 0.026), and transport costs (*p* = 0.006).

**Conclusions:** Compared with conventional management, community-based FCH management achieved similar outcomes, improved psychological health, reduced medical budgets, and thus had a good social prospect.

## Introduction

In recent years, the incidence of chronic kidney disease (CKD) is increasing, CKD now has become one of the major global public health problems [1–3]. The World Health Organization (WHO) reported that there were 220 million people worldwide suffer from CKD [4]. It not only reduced the quality of life of patients but also increased the financial burden of individuals and society [5–7].

At present, the most effective treatment for end-stage renal disease (ESRD) is renal replacement therapy (RRT), including peritoneal dialysis (PD), hemodialysis (HD), and kidney transplantation (KT) [8,9]. Of them, PD is a well-accepted home-based therapy modality with many inherent advantages, including prevention of cross-infection, preservation of residual renal function, maintenance of hemodynamics, improved quality of life, and lower cost than hemodialysis [10]. Moreover, PD equipment is easier to operate and suitable for application in the community. Recently, the increased population of the ESRD in the presence of limited dialysis resources highlights the need for strategies to improve the efficiency and scale of PD management.

Since 2014, Shanghai Songjiang District began to adopt a new community-based management model, which focused on the Family-Community-Hospital three-level comprehensive management and aimed to improve the application and efficiency of PD treatment. Songjiang District Central Hospital provided continuing medical education courses on PD technique to senior nephrologists and nurses from neighboring communities. After a standardized PD catheter insertion in the central hospital, patients underwent routine peritoneal dialysis in community PD units and received home care and follow-up by community doctors. Compared to the traditional PD management model, that patients underwent a routine PD treatment and follow-up only in the central hospital, this new model enlarged PD units and provided more treatment opportunity for rural PD patients.

In recent years, health-related quality of life (HRQOL) and medical costs in ESRD treatment have been evaluated in various studies, aiming to explore a more appropriate dialysis policy [11–16]. Given this, we performed a follow-up study to evaluate the dialysis effect, quality of life, and medical costs for two PD management models, the new Family-Community-Hospital three-level comprehensive management model, and the traditional all-course central hospital management model.

## Methods

### Study design and population

This was a prospective cohort study that included 190 ESRD patients who underwent peritoneal dialysis at Shanghai Songjiang District, including Songjiang District Central Hospital and 8 Songjiang community PD units. The PD patients were recruited from 1 January 2016 to 31 August 2019 and followed up until 31 August 2020. The exclusion criteria were as follows: PD patients with an age younger than 18 years, who were undergoing hemodialysis once a week for dialysis adequacy, who previously accepted hemodialysis or kidney transplantation, and who occasionally underwent PD in other districts. According to the Chinese Peritoneal Dialysis Guideline, we adopted standardized surgical catheterization technique 2016 [17]. Exit-site care, catheter management, and peritonitis prevention referred to International Society for Peritoneal Dialysis (ISPD) Guidelines 2016 and 2017 update [18,19]. Peritonitis was defined according to the ISPD guidelines by 2 of the 3 indices: (1) abdominal pain, (2) dialysate leukocyte count >100 cells/μl with at least 50% neutrophils, (3) positive dialysate microbiological culture [18]. The treatment type of peritoneal dialysis was continuous ambulatory peritoneal dialysis (CAPD), with dextrose dialysate (Baxter Healthcare, Guangzhou, China). According to residual renal function (RRF), body surface area (BSA), and peritoneal equilibrium test (PET) of each patient, dialysis prescription adjusted timely, including dialysate concentration (1.5–2.5%) and dosages (6000–10 000 ml).

According to PD patient’s voluntary, they were divided into two groups: the conventional all-course central hospital management model (*n* = 100) and the new Family-Community-Hospital three-level comprehensive management model (*n* = 90). PD patients received the conventional all-course hospital management, who underwent all-course treatment (including PD catheterization, dialysis, training, and follow-up) only in the central hospital, and they were followed up by hospital PD staff once a month through clinic face to face communication or telephone interview. As for PD patients who received the Family-Community-Hospital management, who underwent three-level comprehensive management. (1) Songjiang District Central Hospital was responsible for catheterization surgery; PD education and training to community medical staff and patients; electronic data management; inpatient treatment for those who under severe complications or need dialysis programs adjustment. (2) Community PD units were responsible for routine community-based PD; dialysis tube sterilization and replacement; PD patients retraining, nursing, and family follow-up; electronic data management; ESRD early diagnosis and suggestion. (3) Family members and patients conducted home-based PD and home care. Patients in the FCH group were followed up by community PD staff once a month through clinic face-to-face communication, telephone interview, and home visit. They were also asked to go to the central hospital for a peritoneal equilibration test every 6 months. In case of severe and refractory peritonitis, pipe adjustment, and catheterization surgery, an additional central hospital visit was in need. In addition, center meetings were held every month with each affiliated community PD unit for the purposes of case discussion, management assessment, and technical training. PD physicians and nurses in both community and central hospital had to participate in Shanghai PD standardization training, obtained qualification certificate, and then worked in the PD department.

This study was conducted according to the guidelines of the Helsinki Declaration. Human Research Ethics Committee of Shanghai Songjiang District Central Hospital approved this study (ChiCTR2000033602). Participant consents were obtained.

### Outcome measures and data collection

The primary aims of the study were clinical outcomes of dialysis therapy, including patient mortality, technique failure, peritonitis episodes, and tunnel infection, etc. The secondary outcomes were HRQOL and medical costs evaluation.

Baseline data of the PD patients were collected by medical staff at the time of recruitment, including age, sex, education, body mass index (BMI) and blood pressure (BP), comorbidity, and medical histories. Venous blood samples were collected and tested every 6 months, and the laboratory data were collected, including serum creatinine (Scr), blood urea nitrogen (BUN), hemoglobin (Hb), serum albumin (ALB), total cholesterol (CHO), triglyceride (TG), parathyroid hormone (PTH), serum calcium (Ca), serum phosphorus (P), the clearance rate of urea nitrogen (Kt/V).

At the end of the follow-up, the physical and psychological status of PD patients were evaluated with HRQOL questionnaires, which were based on the Short-Form 36 (SF-36) score analysis [16]. The SF-36 consists of eight domains: PF, physical functioning; RP, role physical; BP, bodily pain; GH, general health; VT, vitality; SF, social functioning; RE, role emotional; and MH, mental health; with two summary components having been constructed to summarize the physical and mental components.

In addition, the total medical costs and the number of medical visits were collected and calculated from the index date to the last day of the follow-up period by each PD department. The annual medical costs/visits were defined as medical costs/visits within the follow-up period divided by follow-up years. Medical costs included direct and indirect medical costs. The direct medical costs included hospitalization costs and outpatient costs, which were collected from the database of Central Hospital and community PD units. Both central hospital and community PD units followed the same Shanghai medical payment standard. The community did not have a PD inpatient ward. Thus, hospitalization costs only referred to PD related admissions in central hospital, which included: (1) costs of CAPD machine and set; (2) costs of medicine and PD solution; (3) administration, physician nursing, and bed fee; (4) intubation and extubation cost of PD catheter; (5) laboratory test and ultrasound examination fee. Outpatient costs referred to both community and hospital clinic costs, which included: (1) costs of CAPD machine and set; (2) costs of medicine and PD solution; (3) physician and nursing fee; (4) laboratory test fee. The indirect medical costs were transportation costs, which were provided by the patients themselves. We used the China Yuan (CNY) as the currency. One dollar was equal to 6.7606 CNY (5-year average exchange rate, from 2016 to 2020).

### Statistical analysis

IBM SPSS 20.0 software (IBM Corp, Armonk, NY, USA) was used for data analysis. Kolmogorov-Smirnov test was used to assess the distribution of variables, and the Levene test was used to evaluate the homogeneity of variance. Continuous variables conforming to a normal distribution were presented as the mean value plus the standard deviation (mean ± SD), while the skewed distributed data were showed as median values with the 25th to 75th percentile intervals. Enumeration data were presented as the number of cases (*n*) and percentage (%). As for normally distributed data, a student’s *t*-test is used for analyzing the differences between two groups, while comparisons among multiple groups were performed using one-way ANOVA. Non-normally distributed data between two groups were assessed by Mann–Whitney *U* test. Differences in proportion between the two groups were calculated using the Chi-square test or Fisher’s exact test. Comparison of laboratory data of follow-up was performed by repeated measurement analysis of variance. The Kaplan-Meier survival curves were drawn for each event of interest (technique survival and patient survival) and the log-rank test was used to compare curves. For all analyses, *p-*values <0.05 were considered to be statistically significant.

## Results

### Characteristics of the PD patients

A total of 190 CAPD patients with mean age 65.7 ± 13.4 years, and 105 (55.3%) men and 85 (44.7%) women completed this cohort study. As showed in Table 1, there were 100 patients in conventional all-course hospital management and 90 in Family-Community-Hospital management. The two groups have similar ages, sex, education, BMI, and blood pressure (*p* > 0.05). Hypertensive nephropathy (26.0%), diabetic nephropathy (36.0%), and glomerulonephritis (29.0%) were the main reasons for ESRD in the conventional all-course hospital group, while glomerulonephritis (73.3%) was the primary cause of ESRD in the Family-Community-Hospital group. And the number of diabetes mellitus and dyslipidemia in the conventional hospital group was higher than the Family-Community-Hospital group. Both groups suffered similar morbidity of cardiovascular and cerebrovascular diseases.

**Table 1.** Baseline characteristics of two PD management models.

	Total *n* = 190	Conventional management *n* = 100	FCH three-level management *n* = 90	*p*
Male, *n* (%)	105 (55.3)	60(60.0)	45(50.0)	0.166
Age (years)	65.7 ± 13.4	65.5 ± 13.3	65.8 ± 13.6	0.877
BMI (kg/m^2^)	23.1 ± 3.2	23.4 ± 3.5	22.7 ± 2.8	0.126
SBP(mmHg)	147.5 ± 22.1	147.0 ± 22.7	148.0 ± 21.6	0.742
DBP(mmHg)	81.0 ± 12.9	80.2 ± 12.8	81.9 ± 13.1	0.378
Education, *n* (%)				0.129
Illiteracy	36 (19.0)	14 (14.0)	22 (24.7)	
Primary school	60 (31.7)	30 (30.0)	30 (33.7)	
Junior high school	66 (34.9)	40 (40.0)	26 (29.2)	
Senior high school	21 (11.1)	14 (14.0)	7 (7.9)	
Undergraduate	6 (3.2)	2 (2.0)	4 (4.5)	
Cause of ESRD, *n* (%)				<0.001
Hypertensive nephropathy	32 (16.8)	26 (26.0)	6 (6.7)	
Diabetic nephropathy	51 (26.8)	36 (36.0)	15 (16.7)	
Glomerulonephritis	95 (50.0)	29 (29.0)	66 (73.3)	
Hyperuricemic nephropathy	3 (1.6)	0 (0.0)	3 (3.3)	
Other	9 (4.7)	9 (9.0)	0 (0.0)	
Complication, *n* (%)				
Hypertension	187 (98.4)	98 (98.0)	89 (98.9)	0.624
Diabetes mellitus	78 (41.1)	49 (49.0)	29 (32.2)	0.019
Dyslipidemia	78 (41.1)	52 (52.0)	26 (28.9)	0.001
Cardiovascular disease	62 (32.6)	34 (34.0)	28 (31.1)	0.672
Cerebrovascular disease	26 (13.7)	18 (18.0)	8 (8.9)	0.068
Medications n(%)				
CCB	152 (80.0)	78 (78.0)	74 (82.2)	0.468
ARB/ACEI	126 (66.3)	68 (68.0)	58 (64.4)	0.605
Lipid-lowering medications	67 (35.3)	44 (44.0)	23 (25.6)	0.008
Anti-diabetic medications	10 (5.3)	4 (4.0)	6 (6.7)	0.430
Insulin	47 (24.7)	31 (31.0)	16 (17.8)	0.035
Antiplatelet medications	47 (24.7)	29 (29.0)	18 (20.0)	0.151
Calcitriol	43 (22.6)	20 (20)	23 (25.6)	0.361

PD: peritoneal dialysis; FCH: Family-Community-Hospital; BMI: body mass index; SBP: systolic blood pressure, DBP: diastolic blood pressure; ESRD: end-stage renal disease; CCB: calcium channel blocker; ARB/ACEI: angiotensin receptor blocker/angiotensin converting enzyme inhibitors. Other reasons for ESRD included IgA nephropathy, polycystic kidney, obstructive nephropathy.

### Clinical outcomes of PD therapy

To evaluate the therapeutic effect of the Family-Community-Hospital three-level management model of PD, we investigated the outcomes of PD patients under two groups. As showed in Table 2, the median duration of PD in total participation was 43.5 months (interquartile range 26.0–64.3 months). There was no significant difference between conventional all-course hospital management at 41.0 months (23.8–61.8 months) and Family-Community-Hospital management at 45.5 months (26.8–68.0 months) (*p* = 0.606). Among the total participate, 91 (47.9%) PD patients were dropped out. Of the dropout patients, 69 (75.8%) patients died, 3 (3.3%) were converted to renal transplantation therapy and 16 (17.6%) were transferred to hemodialysis, 3 (3.3%) failed to follow-up for some other reasons. As for these death incidents, quite a few patients died of infection 16 (23.2%), cardiovascular disease 11 (15.9%), and cerebrovascular disease 9 (13.0%). The dropout rate and cause of death in the two groups were similar (*p* = 0.366 and *p* = 0.757, respectively). The peritonitis rate in the conventional group was 0.21 episodes per patient year. 52 (52.2%) PD patients had 0 peritonitis episodes, 11 (11.0%) underwent more than 3 times peritonitis episodes during follow-up. The peritonitis rate in the Family-Community-Hospital group was 0.16 episodes per patient year. 46 (51.1%) PD patients had 0 peritonitis episodes, 3 (3.3%) underwent more than 3 times peritonitis episodes during follow-up. The median time to 1st peritonitis episode was 16.5 months in the conventional group, 12.0 in the FCH group (*p* = 0.734).

**Table 2.** Outcomes of PD patients under different management models.

	Total *n* = 190	Conventional management *n* = 100	FCH three-level management *n* = 90	*p*
Duration on PD (months)	43.5 (26.0–64.3)	41.0 (23.8–61.8)	45.5 (26.8–68.0)	0.606
Dropout by cause, *n* (%)				
Overall	91 (47.9)	51 (51.0)	40 (44.4)	0.366
Death	69 (75.8)	38 (74.5)	31 (77.5)	
Kidney transplantation	3 (3.3)	2 (3.9)	1 (2.5)	
Transfer to hemodialysis	16 (17.6)	9 (17.6)	7 (17.5)	
Loss to follow-up	3 (3.3)	2 (3.9)	1 (2.5)	
Cause of death, *n* (%)				0.757
Cerebrovascular disease	9 (13.0)	7 (18.4)	2 (6.5)	
Cardiovascular disease	11 (15.9)	5 (13.2)	6 (19.4)	
Infection	16 (23.2)	8 (21.1)	8 (25.8)	
Electrolyte disorder	5 (7.2)	3 (7.9)	2 (6.5)	
Cachexia	5 (7.2)	3 (7.9)	2 (6.5)	
Other	23 (33.3)	12 (31.6)	11 (35.5)	
Tunnel infection, *n* (%)	8 (4.2)	4 (4.0)	4 (4.4)	0.879
Peritonitis rate (per patient-year)	0.19	0.21	0.16	0.965
Time to 1st peritonitis episode (month)	13.0 (8–24.0)	16.5 (8.3–23.0)	12.0 (7.0–26.8.0)	0.734
Peritonitis episodes, *n* (%)				0.116
0	98 (51.6)	52 (52.0)	46 (51.1)	
1	61 (32.1)	31 (31.0)	30 (33.3)	
2	17 (8.9)	6 (6.0)	11 (12.2)	
≥3	14 (7.4)	11 (11.0)	3 (3.3)	

PD: peritoneal dialysis; FCH: Family-Community-Hospital; Other reasons for death included hypoglycemia, gastrointestinal hemorrhage, fracture, suicide, Alzheimer’s Disease.

**Figure 1.**
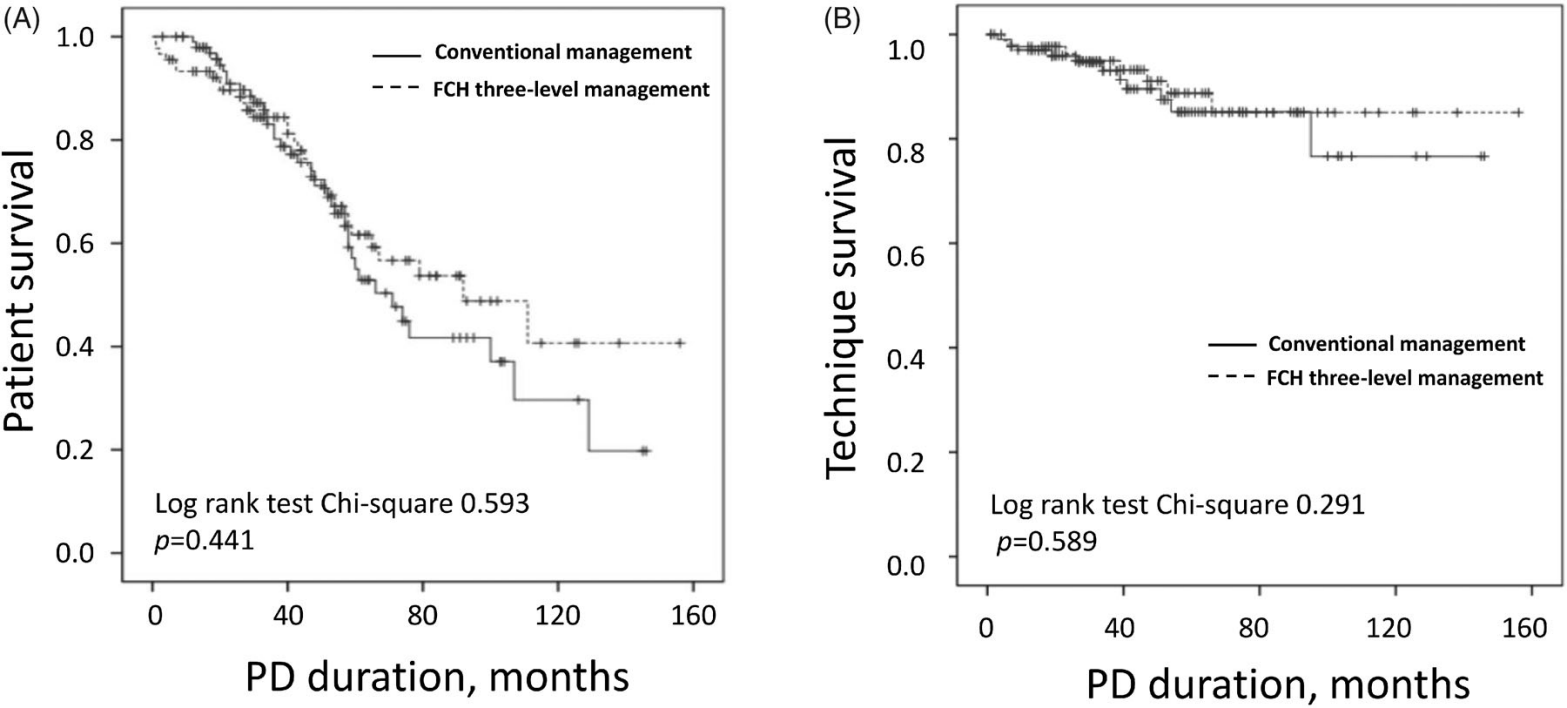
Patient and technique survival in PD patients under two management models. (A) Patient survival. (B) Technique survival. PD: peritoneal dialysis; FCH: Family-Community-Hospital.

Kaplan–Meier analysis showed that the patient survival and technique survival had no significant difference between the two groups (Log-rank 0.593, *p* = 0.441 and Log-rank 0.291, *p* = 0.589, respectively, Figure 1(A,B)). Cox proportional hazards analysis also indicated that different management models hardly impacted the clinic outcomes (hazard ratio (HR) for patient mortality 0.828, 95% confidence interval (CI) 0.515–1.331, *p* = 0.436; HR for technique failure 0.753, 95%CI 0.303–1.874, *p* = 0.542, Supplemental Table1). After confounders adjustment, increased serum albumin was a protective factor for patient survival (HR 0.874, 95%CI 0.813–0.940, *p* < 0.001). And longer time to 1st peritonitis and higher triglyceride level were protective factors for technique survival (HR 0.904, 95%CI 0.822–0.994, *p* = 0.037; HR 0.302, 95%CI 0.906–0.947, *p* = 0.040, respectively).

**Figure 2.**
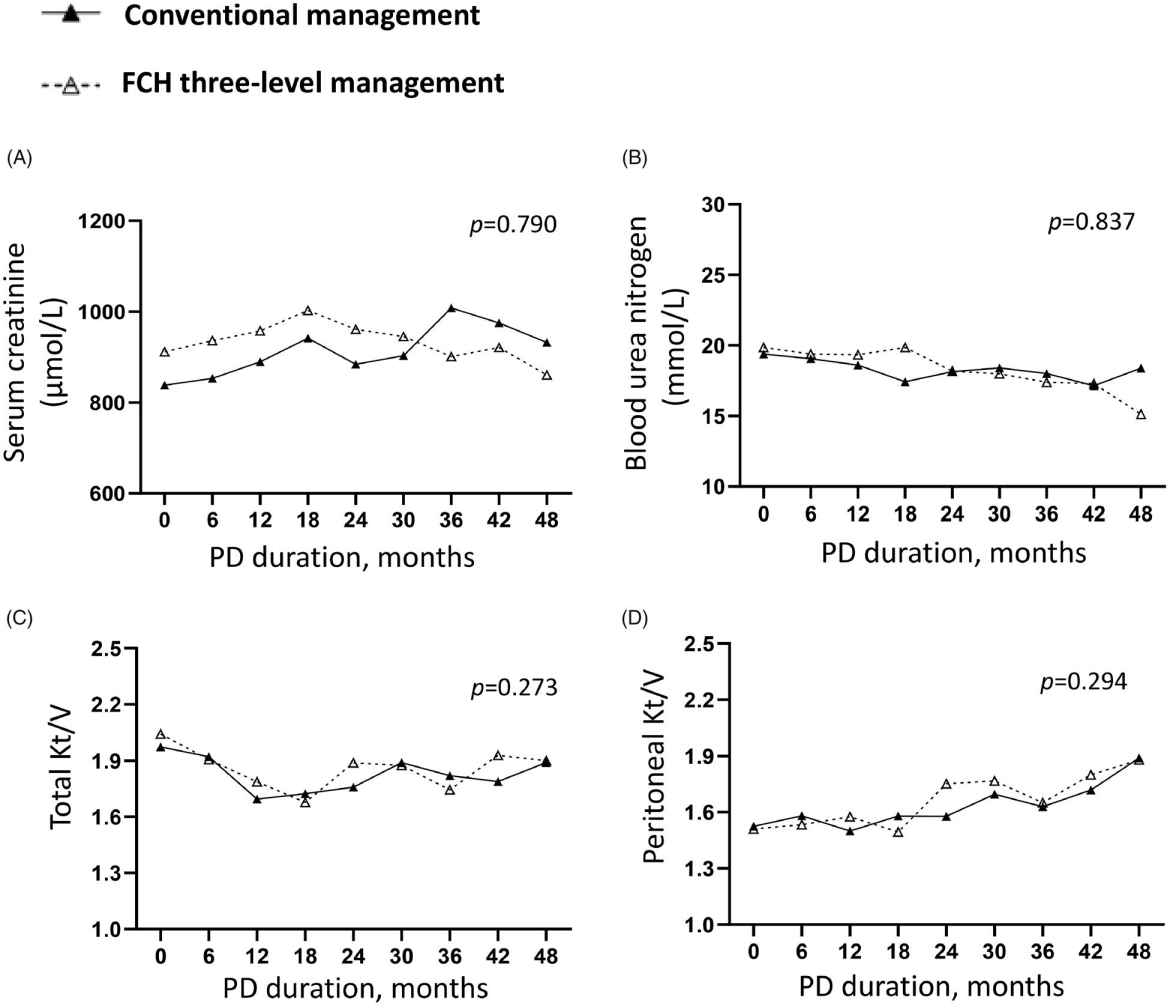
Renal and peritoneal functions in PD patients under two management models. (A) Serum creatinine. (B) Blood urea nitrogen. (C) Total Kt/V. (D) Peritoneal Kt/V. PD: peritoneal dialysis; FCH: Family-Community-Hospital; Kt/V: the clearance rate of urea nitrogen.

**Figure 3.**
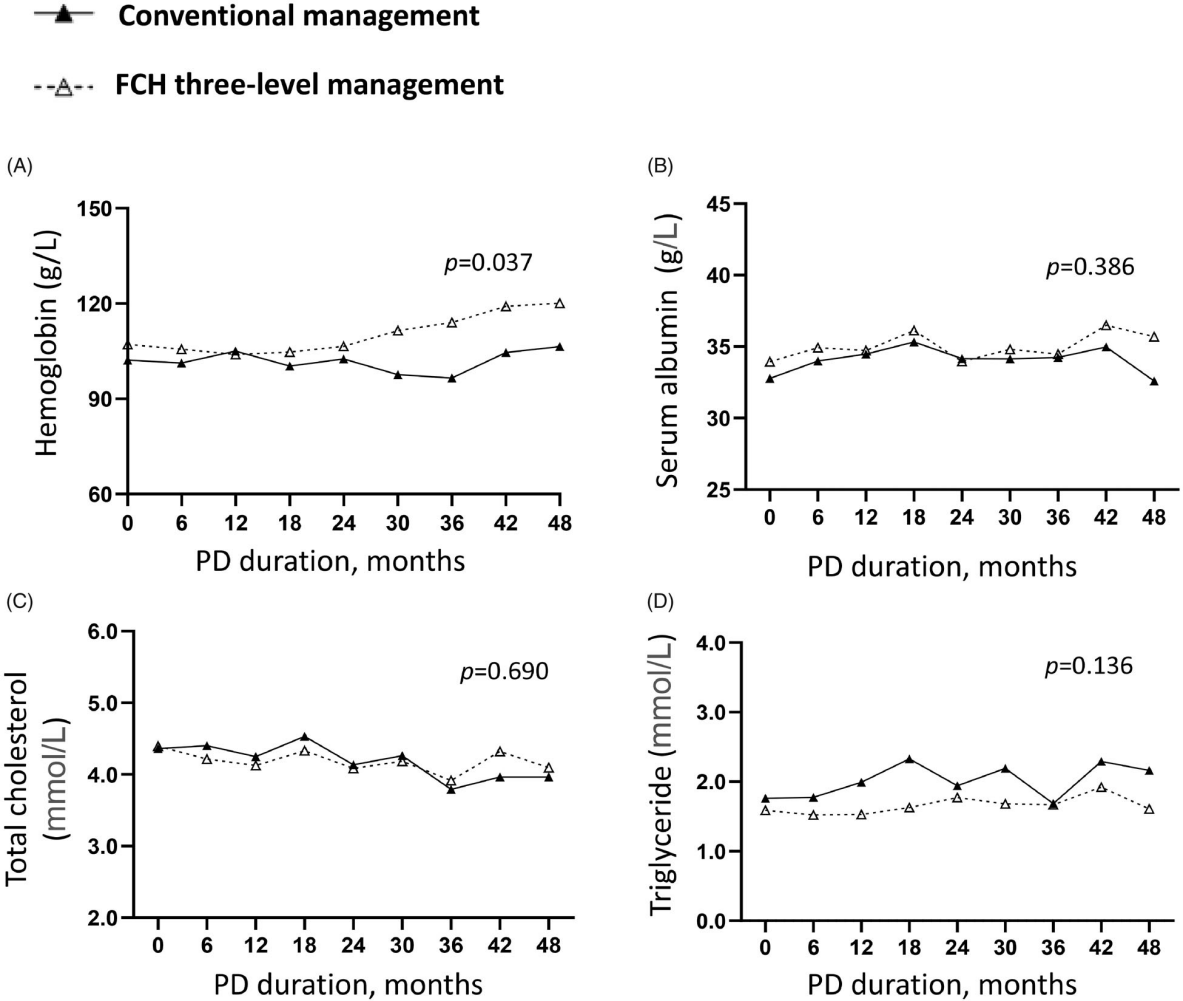
Serum protein and lipid levels in PD patients under two management models. (A) Hemoglobin. (B) Serum albumin. (C) Total cholesterol. (D) Triglyceride. PD: peritoneal dialysis; FCH: Family-Community-Hospital.

**Figure 4.**
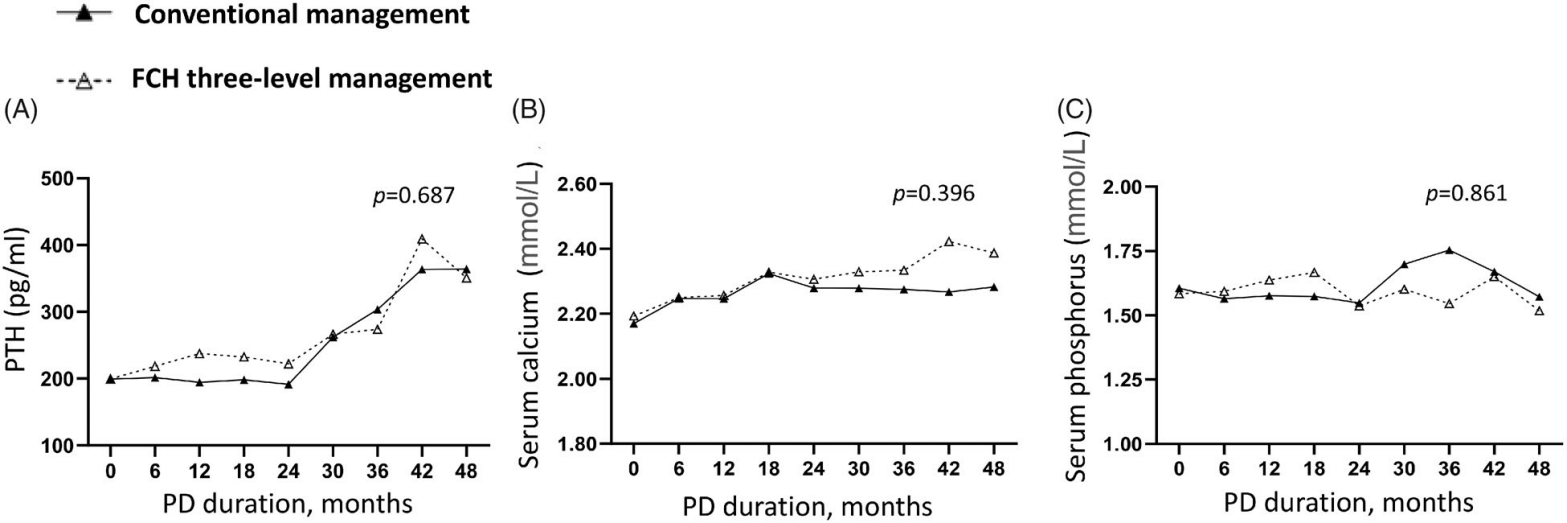
Serum calcium and phosphorus levels in PD patients under two management models. (A) PTH. (B) Serum calcium. (C) Serum phosphorus. PD: peritoneal dialysis; PTH: parathyroid hormone. FCH: Family-Community-Hospital.

In addition, we analyzed the clinic data during the follow-up. Repeated measurement analysis of variance indicated that similar levels of the renal (Figure 2(A,B)) and peritoneal functions (Figure 2(C,D)), serum albumin (Figure 3(B)), cholesterol (Figure 3(C)) and triglyceride (Figure 3(D)), PTH (Figure 4(A)), serum calcium (Figure 4(B)) and phosphorus (Figure 4(C)) in two group (*p* > 0.05). Compared to the conventional group, the Family-Community-Hospital group had a higher hemoglobin level (Figure 3(A), *p* = 0.037). Collectively, these results indicated the Family-Community-Hospital three-level model can achieve a similar dialysis treatment effect as the all-course central hospital model.

### Health-related quality of life

Recently, Short-Form 36 has been widely used to assess the health-related quality of life in dialysis patients [16,20,21]. It is a multicultural scale consisting of 36 questions and categorized into an eight-domain profile of scores: physical functioning, general health, role physical, bodily pain, social functioning, vitality, role emotional, and mental health [22–24]. For each domain, a score ranging from 0 to 100 was assessed with a higher score indicating better health [16]. According to that, SF-36 questionnaires were evaluated at the end of the follow-up. Except for dropout patients (death, transplantation, transfer to hemodialysis, and others), 49 conventionally managed patients and 50 Family-Community-Hospital managed patients participated in the survey. As showed in Table 3, the two groups got a similar score in physical functioning, role-physical, bodily pain, general health, and vitality. While Family-Community-Hospital management achieved a higher score of social functioning, role-emotional, mental health than conventional management (*p* = 0.006, 0.032, and 0.036 respectively). Collectively, these results indicated that the Family-Community-Hospital management model helped to improve the psychological status of PD patients.

**Table 3.** The score of health survey in two PD management models.

	Conventional management *n* = 49	FCH three-level management *n* = 50	*p*
Physical functioning	58.7 ± 13.9	61.2 ± 14.9	0.385
Role-physical	65.3 ± 15.2	68.0 ± 15.2	0.379
Bodily pain	76.3 ± 22.2	74.8 ± 28.6	0.780
General health	39.4 ± 13.1	41.2 ± 16.2	0.556
Vitality	44.6 ± 18.2	45.9 ± 17.1	0.713
Social functioning	48.0 ± 20.1	58.8 ± 18.3	0.006
Role-emotional	55.1 ± 23.1	66.0 ± 26.5	0.032
Mental health	46.5 ± 12.7	52.4 ± 14.9	0.036

PD: peritoneal dialysis; FCH: Family-Community-Hospital.

### Medical cost evaluation

**Figure 5.**
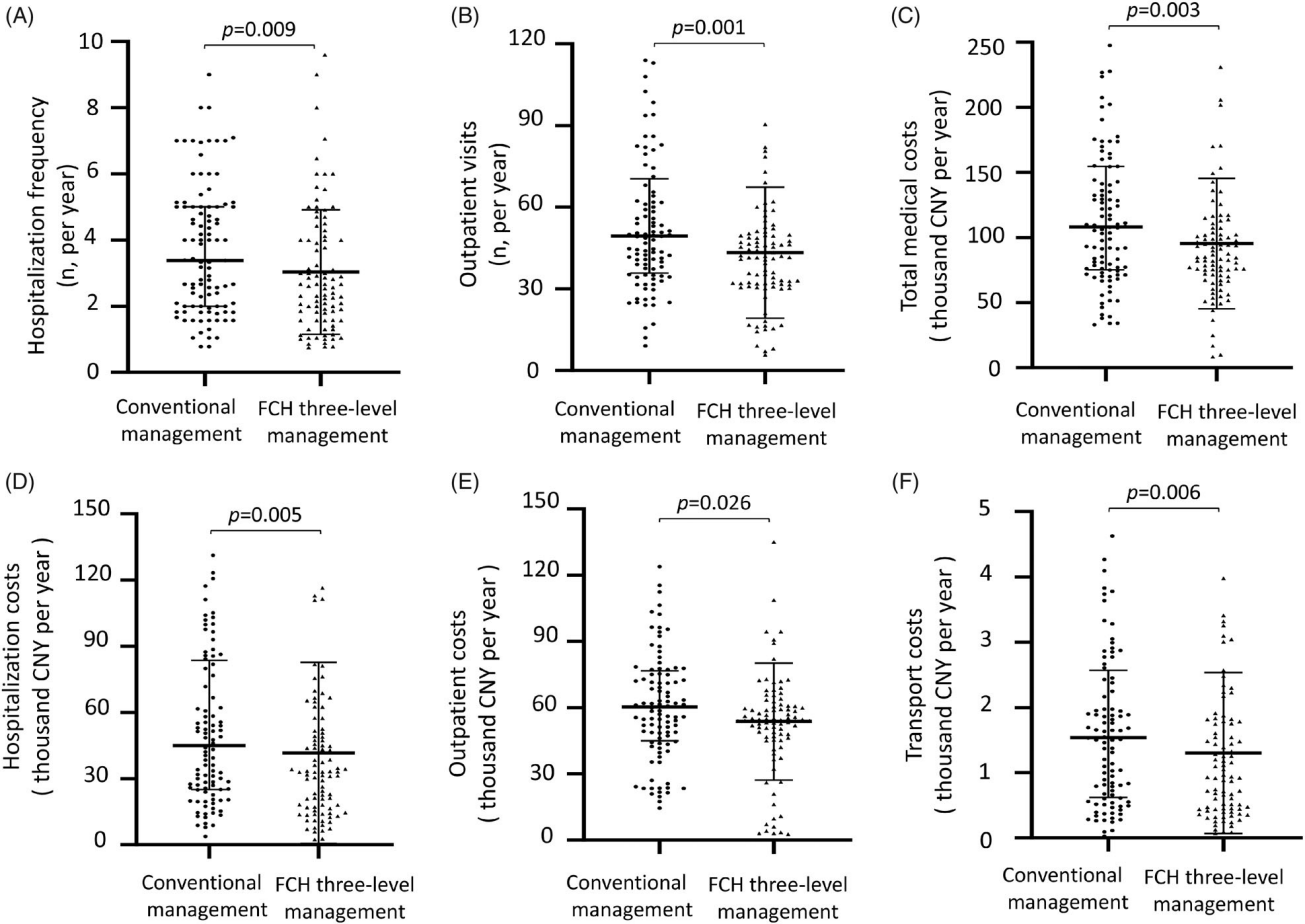
The annual medical visits and costs of two management models. (A) Hospitalization frequency. (B) Outpatient visits. (C) Total medical costs. (D) Hospitalization costs. (E) Outpatient costs. (F) Transport costs. Hospitalization costs and visits only refer to PD related admissions in central hospital. Community do not have PD inpatient ward. Outpatient costs and visits refer to both community clinic and central hospital clinic. Transport costs refer to the costs only payed for commuting to central hospital. PD: peritoneal dialysis; FCH: Family-Community-Hospital; CNY: China Yuan. One dollar was equal to 6.7606 CNY (5 year average exchange rate, from 2016 to 2020).

To evaluate the financial budget of the two management groups, we recorded the annual medical costs, which included direct and indirect medical costs. The direct medical costs included hospitalization costs and outpatient costs, indirect medical costs were transportation costs. As showed in Table 4 and Figure 5, the frequency of annual hospitalizations and clinics in the Family-Community-Hospital group was less than that in the conventional group (Figure 5(A,B), *p* = 0.009, *p* = 0.001, respectively). In addition, compared to conventional management, Family-Community-Hospital management also had lower annual hospitalization costs (Figure 5(D), *p* = 0.005), outpatient costs (Figure 5(E), *p* = 0.026), total medical costs (Figure 5(C), *p* = 0.003) and transport costs (Figure 5(F), *p* = 0.006). These results indicated that the new Family-Community-Hospital three-level comprehensive management model reduced the medical budget and had a good prospect.

**Table 4.** The annual expenses of two PD management models.

	Conventional management *n* = 100	FCH three-level management *n* = 90	*p*
Hospitalization frequency (*n*)	3.4 (2.0–5.0)	2.6 (1.6–4.0)	0.009
Outpatient visits (*n*)	49.5 (35.8–70.4)	41.8 (31.7–49.7)	0.001
Hospitalization expenses (CNY)	44 995.8 (25 057.8–83 557.8)	32 978.6 (16 764.1–50 926.9)	0.005
Outpatient expenses (CNY)	60 299.0 (45 025.6–76 704.2)	54 492.3 (46 027.5–61 640.3)	0.026
Total medical expenses (CNY)	108 108.5 (75 204.3–154 539.8)	85 872.7 (70 554.9–107 299.5)	0.003
Transport expenses (CNY)	1 538.0 (623.6–2 567.0)	951.6 (442.6–1 783.0)	0.006
Distance (kilometer)	9.1 (3.7–15.8)	11.0 (3.5–14.3)	0.353

PD: peritoneal dialysis; FCH: Family-Community-Hospital. CNY: China Yuan. One dollar is equal to 6.4489 CNY. Hospitalization expenses and visits only refer to PD related admissions in central hospital. The community does not have a PD inpatient ward. Outpatient expenses and visits refer to both community clinic and central hospital clinic. Transport expenses refer to the costs only payed for commuting to central hospital. Distance refers to the distance from home to central hospital.

## Discussion

In this prospective cohort study, we provided a clinical outcome, quality of life, and costs evaluation of the Family-Community-Hospital three-level management model of PD in Shanghai Songjiang District. Our research indicated that Family-Community-Hospital management achieved a similar dialysis treatment effect as the conventional all-course central hospital model. Moreover, this new model improved the psychological status and saved the medical costs of PD patients. Considering the advantage of the enlargement of PD resources, this comprehensive management model has a good societal prospect.

Currently, attendance at an uniform training program at Shanghai District Central Hospital ensured that well-trained PD nephrologists and nurses were available in its affiliated community PD units. All the patient management procedures were based on guidelines established by the ISPD and the U.S. National Kidney Foundation’s Kidney Disease Outcomes Quality Initiative [25–28]. Compared with conventional management, this kind of community-based management enlarged the scales of PD treatment and met the need of increased ESRD patients. Our research showed that the new Family-Community-Hospital three-level comprehensive management model achieved a similar dialysis treatment effect as the conventional model. Follow-up data showed that similar levels of the renal and peritoneal functions, serum albumin, cholesterol and triglyceride, PTH, serum calcium, and phosphorus between the two groups. There was also no significant difference in drop-out rate, peritonitis rate, patient survival, and technique survival. Similarly, there was also an exploration of the PD management model in other regions of China. For instance, the First Affiliated Hospital of the Sun Yat-sen University established a PD satellite center program in Guangdong province [29]. Their First Affiliated Hospital was the central hospital for peritoneal dialysis, satellite PD units were selected in various areas across Guangdong province. Notably, their clinical effects had remarkably improved in the satellite PD units. The annual drop-out rate had decreased from 28.2% to 18.2%, the average rate of peritonitis in the study had declined to 1 episode in 54.5 patient-months from 1 episode in 39.4 patient-months, and the 1-year patient survival rate increased from 82.0% to 84.2% [30]. Compared with their lower drop-out rate and higher survival rate, our drop-out rate was 47.9%, the survival rate was 63.6%. If death was censored for technique failure, our present technique survival rate was 90.0% (171/190), which was equivalent to Guangdong province. To find the reasons, we recorded the outcomes of each patient. Of the 190 PD patients, 91 patients dropped out. 69 patients died, 3 patients received kidney transplantation, 16 patients transferred to hemodialysis, 3 patients failed to follow-up. Death was the main reason for higher drop-out and lower survival rates. Notably, average age in Guangdong PD center was 48.1, while 65.7 in our Shanghai Songjiang district. Because of the unbalanced regional development and the aged tendency of the Chinese population, different PD centers had patients under different age phases. Senior citizens accounted for the main part of PD population in Shanghai Songjiang. The elder patients were vulnerable to suffer cardio-cerebrovascular complications, infection even death. To reducing the death-induced dropout, we decided to improve the nursing and monitoring to potentially lethal complications. Complication management might be out of the dialysis field, while it was indeed important for elder PD patients’ outcomes. We were continuously exploring the PD management models to improve the survival rate, therapeutic efficiency and expand the PD resources. Although successful PD management experience was worth learning, PD satellite research did not address their issue of PD costs.

Our study recorded the annual medical costs of different management groups and further analyzed their economic effectiveness. We found that the Family-Community-Hospital management model was more cost-effective than the conventional model as its lower costs, including direct medical costs (the annual hospitalization costs and outpatient costs) and indirect medical costs (transport costs). Generally, the indirect costs included the transportation costs for commuting to the central hospital, additional water and electricity costs in case of home dialysis, nutrition management, nursing fee, the productivity loss of patients and their caregivers [31]. However, it was difficult to evaluate the individual economic differences of each family and other related factors. We did not take these costs into account, the indirect medical costs only recorded transportation costs. Given that the distance to the central hospital was similar in the two groups, reduced transport costs might attribute to a decreased commuting frequency based on the hospitalization and outpatient visits. FCH model had multiple follow-up forms, especially for a home visit. A home visit by the PD nurse was often useful in detecting problems with exchange technique, adherence to protocols, and other environmental and behavior issues, which reduced the out-patient consultation and in-patient treatment for some PD-related complications. It not only reduced the toil of the route, but also decreased the medical costs of individuals and government, and thus had a good societal prospect.

Patients with ESRD will be more or less affected in mood, emotional function, and social function [32,33]. Severe patients can develop depression, even independent of dialysis mode [34,35]. Improving the quality of life of PD patients was a big challenge in China. Our results from the HRQOL survey showed that the Family-Community-Hospital management model helped to improve the psychological status of PD patients, such as social functioning, role emotional, and mental health. It might attribute to the high frequency of home follow-up and warm humanistic care from community doctors and the convenience from community/home treatment. In fact, more and more chronic diseases, such as diabetes, hypertension, chronic obstructive pulmonary disease, had gradually begun to implement a community graded management model for disease administration and had proved to be beneficial to patients [36,37]. For instance, Bruce et al.[37] demonstrated that the community PD units improved care by an additional 4.5 ∼ 4.9 percentage points as compared with controls. The community PD units also had remarkable improvement for prevention with an increase of 4.5 ∼ 6.2 percentage points. The disease monitoring and treatment also significantly improved with an increase of 5.5 ∼ 5.9 percentage points. Similarly, Carrie et al.[36] indicated that implementation of community-based health improvement programs was related to the decrease of the rate of obesity, a decrease in the proportion of people reporting being in poor health, and a smaller increase in the rate of smoking. This study provided evidence for the important role of the communities in improving the psychological health of patients.

Some limitations of the study should be acknowledged. Firstly, patients were divided into two groups by their own voluntary, not by commute distance or blind method. Because this was a pilot project for PD management in Songjiang. Many patients in remote areas still insisted on receiving conventional management in the central hospital regardless of the inconvenient commute. Even so, most of the baseline characteristics of the two groups had no statistical difference, except for diabetes, dyslipidemia, drugs for diabetes, and dyslipidemia, cause of ESRD. In the Cox proportional-hazards for outcomes, the differences in diabetes and dyslipidemia had no significant influence on patient mortality and technique failure. Thus, the bias caused by self-selection was limited, and our results were tenable. Secondly, the sample size of our study was relatively small and with quite a few death-induced dropouts, further larger size and follow-up investigation are necessary. Thirdly, the present data lacked the detail in the family economy and living styles. Fourthly, more indirect medical costs were not available, such as nutrition management, nursing fee, etc. Nonetheless, the strengths of our study included its strict exclusion criteria based on medical histories, and a multi-center study was conducted.

In conclusion, Family-Community-Hospital three-level comprehensive management model achieved a similar therapeutic effect as the all-course central hospital management model, improved psychological health, reduced treatment budgets, and thus had a good social prospect.

## Supplementary Material

Supplemental Material

## Data Availability

The data sets generated and analyzed during the current study are available from the corresponding author upon reasonable request.

## Abbreviations

ALB: albumin
ARB/ACEI: angiotensin receptor blocker/angiotensin converting enzyme inhibitors
BMI: body mass index
BP: blood pressure
BSA: body surface area
BUN: blood urea nitrogen
Ca: calcium
CAPD: continuous ambulatory peritoneal dialysis
CCB: calcium channel blocker
CHO: cholesterol
CI: confidence interval
CKD: chronic kidney disease
CNY: China Yuan
DBP: diastolic blood pressure
ESRD: end-stage renal disease
FCH: Family-Community-Hospital
Hb: hemoglobin
HD: hemodialysis
HR: hazard ratio
HRQOL: health-related quality of life
ISPD: International Society for Peritoneal Dialysis
KT: kidney transplantation
Kt/V: the clearance rate of urea nitrogen
P: phosphorus
PD: peritoneal dialysis
PET: peritoneal equilibrium test
PTH: parathyroid hormone
RRF: residual renal function
RRT: renal replacement therapy
SBP: systolic blood pressure
SF-36: Short-Form 36
Scr: serum creatinine
TG: triglyceride
WHO: World Health Organization
