# Supplementary material for: Clinical outcomes, quality of life, and costs evaluation of peritoneal dialysis management models in Shanghai Songjiang District: a multi-center and prospective cohort study
Source: Ren Fail. 2021 Apr 29;43(1):754–65. doi: 10.1080/0886022X.2021.1918164 (PMC8901286; doi:10.1080/0886022X.2021.1918164)
Supplement: Supplemental Material [file IRNF_A_1918164_SM9226.pdf]

Supplemental table1. Cox proportional-hazards for patient mortality and technique failure.

	Univariate Cox regression analysis			Multivariate Cox regression analysis		
	HR	95%*CI*	*P*	*HR*	95%*CI*	*P*
**Patient mortality**						
Management model (FCH vs. control)	0.828	0.515-1.331	0.436			
Male (male vs. female)	1.007	0.627-1.615	0.978			
Age (years)	0.987	0.969-1.004	0.134			
BMI (kg/m^2)	1.001	0.935-1.071	0.981			
Time to first peritonitis (months)	1.009	0.992-1.028	0.299			
Hemoglobin (g/L)	0.971	0.954-0.989	0.001	0.982	0.962-1.003	0.088
Serum albumin (g/L)	0.855	0.808-0.904	<0.001	0.874	0.813-0.940	<0.001
Total cholesterol (mmol/L)	1.061	0.787-1.429	0.699			
Triglyceride (mmol/L)	1.060	0.833-1.348	0.636			
Total Kt/V	0.263	0.096-0.722	0.010	0.502	0.171-1.470	0.209
Peritoneal Kt/V	0.560	0.239-1.312	0.182			
Hypertension (yes vs. no)	20.942	0.024-18388.357	0.379			
Diabetes mellitus (yes vs. no)	1.243	0.774-1.996	0.367			
Dyslipidemia (yes vs. no)	1.452	0.905-2.328	0.122			
Cardiovascular disease (yes vs. no)	1.242	0.763-2.021	0.383			
Cerebrovascular disease (yes vs. no)	1.642	0.913-2.952	0.098			
**Technique failure**						
Management model (FCH vs. control)	0.753	0.303-1.874	0.542			
Male (male vs. female)	2.594	0.933-7.208	0.068			
Age (years)	0.991	0.957-1.025	0.584			
BMI (kg/m^2)	0.952	0.832-1.088	0.468			
Time to first peritonitis (months)	0.888	0.804-0.982	0.020	0.904	0.822-0.994	0.037
Hemoglobin (g/L)	0.981	0.947-1.015	0.269			
Serum albumin (g/L)	0.965	0.869-1.071	0.505			
Total cholesterol (mmol/L)	0.697	0.370-1.314	0.265			
Triglyceride (mmol/L)	0.252	0.100-0.632	0.003	0.302	0.906-0.947	0.040
Total Kt/V	0.767	0.149-3.941	0.751			
Peritoneal Kt/V	0.356	0.081-1.567	0.172			
Hypertension (yes vs. no)	20.922	0.000-8804084.171	0.645			
Diabetes mellitus (yes vs. no)	0.848	0.334-2.155	0.729			
Dyslipidemia (yes vs. no)	0.370	0.123-1.116	0.078			
Cardiovascular disease (yes vs. no)	0.560	0.186-1.689	0.303			
Cerebrovascular disease (yes vs. no)	0.360	0.048-2.697	0.320			

The reason for wide CI for hypertension may attribute to the very small number of unexposed hypertension cases 2/100 in conventional group, and 1/90 in FCH group.
